# B-type natriuretic peptide changes and left ventricular remodeling dynamics in heart failure with reduced ejection fraction

**DOI:** 10.3389/fcvm.2025.1666067

**Published:** 2026-01-08

**Authors:** Erick Romero, Yevhen Kushnir, Areej Shahzad, Dev Jaydeep Patel, Bang Heejung, Padmini Sirish, Nipavan Chiamvimonvat, David A. Liem, Martin Cadeiras

**Affiliations:** 1 Department of Internal Medicine, St. Barnabas Hospital, SBH Health System, City University of New York (CUNY) School of Medicine, Bronx, NY, United States; 2 Division of Cardiovascular Medicine, Department of Internal Medicine, University of California, Davis, Davis, CA, United States; 3 Division of Biostatistics, Department of Public Health Sciences, University of California Davis, Davis, CA, United States; 4 Division of Cardiovascular Medicine, UC Davis Medical Center, Sacramento, CA, United States

**Keywords:** BNP, HFrEF, LVEF, remodeling, longitudinal

## Abstract

**Background:**

B-type natriuretic peptide (BNP) is an important biomarker in heart failure with reduced ejection fraction (HFrEF). We aimed to explore changes in BNP and their relationship with long-term dynamics of left ventricular (LV) geometry.

**Methods:**

This was a single-center retrospective cohort. Inclusion criteria included LV ejection fraction (LVEF) < 40% measured by echocardiography, BNP ≥100 pg/mL at baseline, and a subsequent BNP measure within a year. Percent BNP change from baseline was computed and divided into tertiles. Percent change tertiles represented decreasing (min—max, −63.3 to −10.4), minimal changes (−10.4 to 2.8), and rising BNP levels (2.9 to 12.6). The study endpoint included LV internal dimension at end-systole (LVIDs), LV internal dimension at end-diastole (LVIDd), and LVEF. The secondary endpoint consisted of all-cause mortality.

**Results:**

A total of 887 patients were included. Baseline characteristics, including age, sex, blood pressure, atrial fibrillation, baseline BNP, and LVEF, varied among tertiles (*p* < 0.05). When comparing to the rising BNP tertile, the decreasing BNP tertile showed decreased trends of LVIDs (*p* = 0.001), LVIDd (*p* = 0.006); and increased trends of LVEF (*p* = 0.008). All-cause mortality was higher in the rising BNP tertile (*p* < 0.05) compared to the decreasing tertile.

**Conclusion:**

In a real-world routine HFrEF cohort, this study demonstrates the *time-dependent* relationship between BNP changes, LV remodeling dynamics, and survival outcomes. Findings contribute to the literature supporting BNP as a dynamic marker for LV remodeling.

## Introduction

B-type natriuretic peptide (BNP) is an important clinical biomarker in heart failure (HF) (1). Elevated BNP levels are strongly indicative of HF, and are commonly utilized in clinical practice to guide management (2). The ventricular myocardium secretes this BNP in response to increased wall stress and pressure overload (3). It is a counter-regulatory hormone that promotes vasodilation, natriuresis, and diuresis (4).

A key pathophysiological process in HF with reduced ejection fraction (HFrEF) is left ventricular (LV) remodeling (5). The LV remodeling is marked by progressive dilation, wall thinning, and reduced contractile function (6). Importantly, LV remodeling correlates with adverse clinical outcomes (7, 8). Thus, LV remodeling is an important marker in HFrEF.

While the relationship between natriuretic peptides and LV remodeling exists, the relationship between changes in BNP and time-dependent LV remodeling has not been extensively studied in real-world settings (6, 9, 10). Therefore, the aim of this study was to evaluate the association between changes in BNP and long term LV remodeling in real-world clinical data. We hypothesized that patients who experience greater reductions in BNP levels would demonstrate more favorable LV remodeling and survival outcomes.

## Methods

### Data source, study design, and inclusion criteria

We conducted a single-center retrospective cohort study using data collected from the University of California, Davis Medical Center. Data was collected through the institution's electronic health record data warehouse using a time frame between January 2014 to December 2022. Inclusion criteria for the HFrEF cohort consisted of adults aged ≥18 years, International Classification of Diseases (ICD) HF codes (ICD-9 or ICD-10), LVEF ≤40%, and BNP ≥100 pg/mL. Exclusion criteria included patients with ICD codes for cardiac transplant or left ventricular assist device implantation, those without follow-up LVEF values, and those with less than a one-year interval between their first and second BNP measurements. The cohort has been previously validated with a specificity of 0.96 and a sensitivity of 0.60 as compared with physician chart review (11).

### Baseline definitions

The cohort index date was defined as the date of the first recorded LVEF ≤40%. Demographic variables included age, sex, race, and ethnicity. Baseline laboratory, echocardiogram, and electrocardiogram characteristics were defined as the first value within 90 days or the first available after the indexation date. Comorbidities were defined as diagnoses prior to the cohort index date, and validated comorbidity identification methodologies were used. For this study, we defined guideline-directed medical therapy (GDMT) utilization as the prescription of medications within 6 months prior to and within 3 months after the cohort indexation date. Medications included renin-angiotensin system inhibitors (RASi), β-blockers, mineralocorticoid receptor antagonists (MRA) and sodium-glucose co-transporter 2 inhibitors (SGLT-2). The RASi category comprised angiotensin-converting enzyme inhibitor, angiotensin receptor blocker, and angiotensin receptor neprilysin inhibitor. Patients were placed into one of three dosing categories based on their recorded prescription: either none, <50%, or ≥50% of GDMT target dosing.

### Longitudinal data and cohort stratification

Longitudinal laboratory, electrocardiogram, and echocardiogram variables were extracted, starting from the cohort indexation date until the last available LVEF measurement. After BNP log transformation, percent BNP changes from baseline at one-year were computed, then the cohort was stratified into low, middle, and high BNP change tertiles.

### Study outcomes

The co-primary outcomes were LV internal dimension at end-systole (LVIDs), LV internal dimension at end-diastole (LVIDd), and LVEF, all measured by echocardiography. The secondary outcome included all-cause mortality. All-cause mortality data was retrieved from the institution's clinical data warehouse. However, cause-specific mortality was not available.

### Statistical analysis

Descriptive statistics were used to summarize baseline characteristics. Between-group comparisons were performed using Wilcoxon signed-rank test for continuous variables or ANOVA, and chi-squared or Fisher exact test for categorical variables as appropriate.

For outcome analysis, linear mixed models were performed to assess changes between BNP change groups and the endpoints over time. An exploratory multinomial logistic regression analysis was conducted to explore GDMT association to BNP tertile changes adjusted for potential factors influencing GTMT use (age, sex, blood pressure, heart rate, atrial fibrillation, hypertension, creatinine and K+). A Cox regression model was constructed for survival analysis adjusting for relevant covariates known to influence BNP levels (age, sex, BMI, atrial fibrillation, baseline BNP, creatinine, and LVEF were included) (12). Analyses were performed using SAS software version 9.4 (SAS institute, Cary, NC, USA).

### Ethics

The university's Institutional Review Board approved the study, and all data used were de-identified before analysis.

## Results

### Cohort identification and BNP change tertile stratification

**Figure 1:**
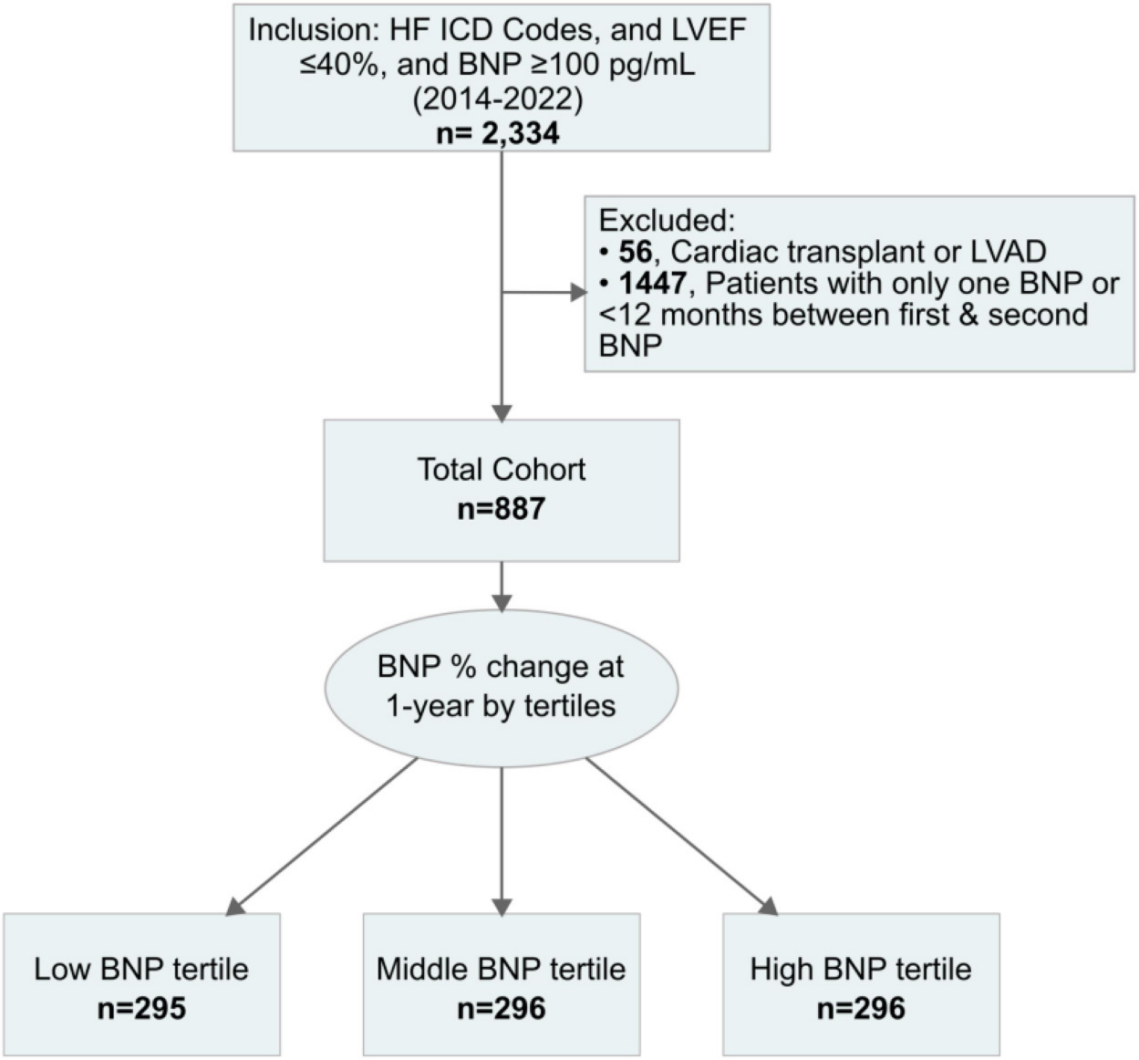
Cohort identification workflow. BNP, B-type natriuretic peptide; HF, heart failure; ICD Codes, international classification of diseases codes; LVAD, left ventricular assist device; LVEF, left ventricular ejection fraction.

**Figure 2:**
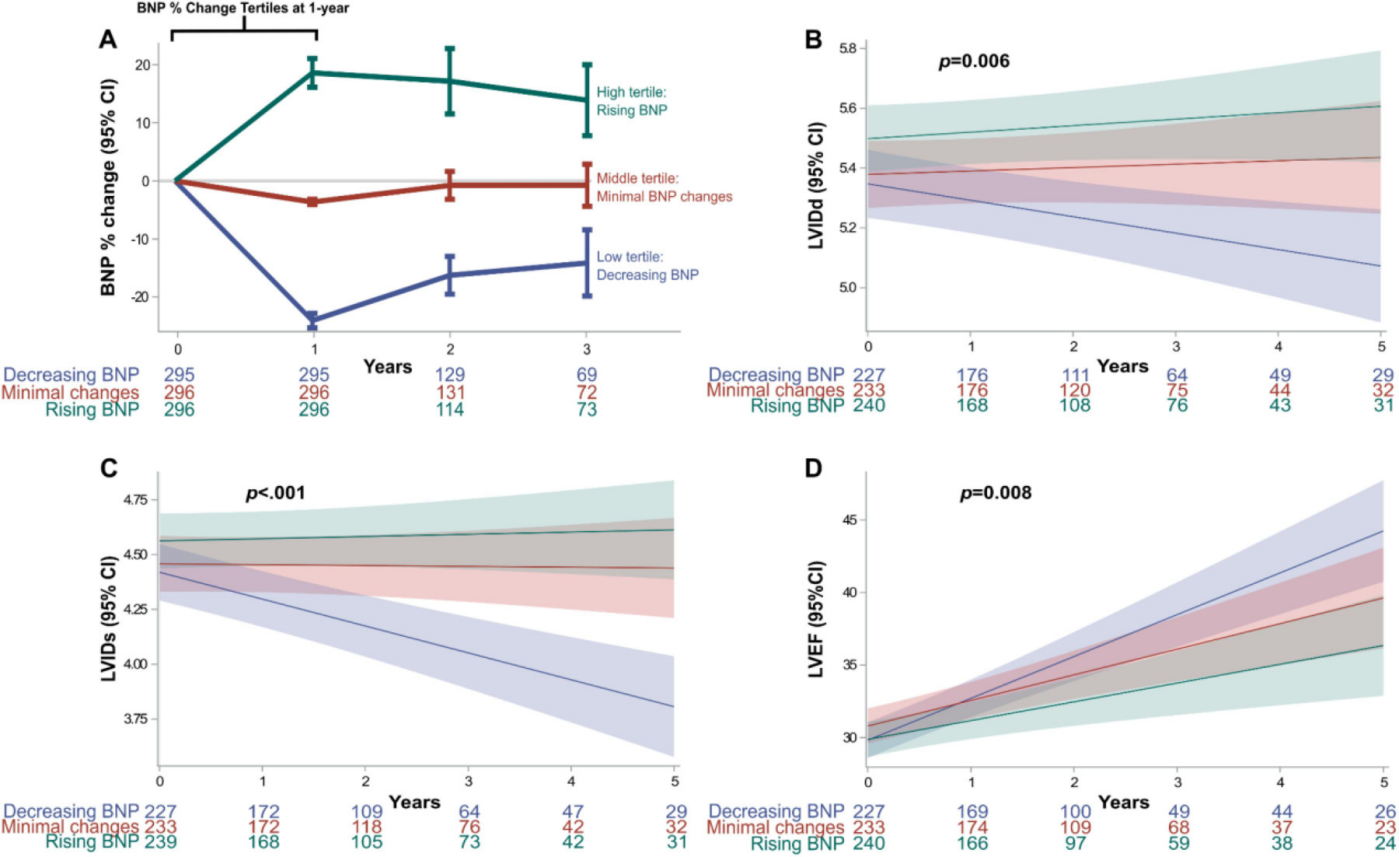
BNP tertile changes and co-primary endpoints. **(A)** Absolute BNP percent change at one-year from baseline: The first year was used to categorize groups based on BNP percent change tertiles. **(B–D)** Co-primary outcomes assessing echocardiographic LV geometry and function showed long-term changes between the BNP change groups. Mixed linear model showed significant differences in LVIDs (*p* < 0.006), LVIDd (*p* < .001) and LVEF (*p* < 0.008). BNP, brain natriuretic peptide; LV, left ventricular; LVEF, left ventricular ejection fraction; LVIDd, LV internal dimension at end-diastole; LVIDs, LV internal dimension at end-systole.

An initial 2,334 patients were included. However, in the final cohort, only 887 cases met the inclusion criteria (Figure 1). Percent changes in BNP at one-year were categorized into tertiles: low tertile (*n* = 295), middle tertile (*n* = 296), and high tertile (*n* = 296). Corresponding to decreasing, minimal changes, and worsening BNP levels (Figure 1). The decreasing BNP tertile had a mean BNP reduction of −24% (95% CI, −25% to −22%); the rising BNP group had a mean increase of 18% (95% CI, 16%–21%); the minimal BNP change tertile showed a decrease of −4% (95% CI, −4% to −3%), see Figure 2A.

### Baseline characteristics

Baseline characteristics varied across BNP tertiles (Table 1). Patients in the decreasing BNP tertile were younger, a median age of 61 years (IQR 53.0–70.5), vs. a median of 65 years (IQR 56.0–77.0) in the rising BNP tertile (*p* < 0.001). Overall, the cohort was predominantly male (68.8%) with a higher male proportion in the minimal changes and rising tertiles groups (73.0% and 69.6%, respectively). Hispanic comprised a 21.4% of the decreasing BNP tertile (*p* < 0.001). Blood pressure and heart rate were elevated in the decreasing BNP tertile group (80.0 mmHg, *p* = 0.022; and 91.0 bpm, *p* = 0.004, respectively). The minimal BNP changes group presented the highest rates of atrial fibrillation patients (41.2%, *p* = 0.007).

**Table 1:** Patient baseline characteristics by tertile percent changes.

Median (IQR) or No. (%)						
Characteristics	Miss	Overall (*n* = 887)	Low tertile, decreasing BNP	Middle tertile, minimal BNP changes	High tertile, rising BNP	*P* *
Tertile min-max			−63.3 to −10.4	−10.4 to 2.8	2.9 to 12.6	
			(*n* = 295)	(*n* = 296)	(*n* = 296)	
Age, years	0	64 (55–75)	61 (53.0–70.5)	65 (57–77)	65 (56–77)	<0.001
Sex	0					0.049
Male		610 (68.8)	188 (63.7)	216 (73.0)	206 (69.6)	
Race	0					0.075
White		506 (57.0)	148 (50.2)	187 (63.2)	171 (57.8)	
Black		159 (17.9)	61 (20.7)	46 (15.5)	52 (17.6)	
Asian		46 (5.2)	13 (4.4)	14 (4.7)	19 (6.4)	
Hawaiian/Native American		23 (2.6)	7 (2.4)	7 (2.4)	9 (3.0)	
Other		146 (16.4)	63 (21.3)	40 (13.5)	43 (14.5)	
Unavailable		7 (0.8)	3 (1.0)	2 (0.7)	2 (0.7)	
Ethnicity	0					<0.001
Non-Hispanic		761 (85.8)	229 (77.6)	271 (91.6)	261 (88.2)	
Hispanic		120 (13.5)	63 (21.4)	24 (8.1)	33 (11.1)	
Unavailable		6 (0.7)	3 (1.0)	1 (0.3)	2 (0.7)	
Vitals/biometrics						
Systolic BP, mmHg	3	125 (111–142)	126 (110–143)	121 (109–139)	128 (113–143.8)	0.100
Diastolic BP, mmHg	3	77 (67–89)	80 (68–92)	76 (66–87)	76 (68–88)	0.022
MAP, mmHg	3	94 (82.7–105.3)	96.7 (82.2–106.3)	90.7 (81.8–103.7)	94 (84.1–105.0)	0.068
Heart Rate, beats/min	1	89 (77–102)	91 (80–105)	88 (76–102)	86 (75–99.2)	0.004
BMI, kg/m^2^	91	28.4 (24.6–33.4)	28.2 (24.9–32.9)	28.7 (24.8–32.8)	28.2 (24.3–33.8)	0.934
Medical history						
Hypertension	8	639 (72.7)	215 (73.4)	201 (69.1)	223 (75.6)	0.198
Diabetes	8	402 (45.7)	130 (44.4)	123 (42.3)	149 (50.5)	0.114
Hyperlipidemia	8	390 (44.4)	123 (42.0)	125 (43.0)	142 (48.1)	0.271
Ischemic Heart Disease	8	325 (37.0)	96 (32.8)	118 (40.5)	111 (37.6)	0.144
Atrial Fibrillation	8	301 (34.2)	86 (29.4)	120 (41.2)	95 (32.2)	0.007
Chronic Kidney Disease	8	244 (27.8)	77 (26.3)	77 (26.5)	90 (30.5)	0.432
Laboratory						
BNP, pg/mL	0	663 (298–1,305)	837 (419–1,553)	698 (362.5–1,344.5)	433.5 (139.2–981.0)	<0.001
NT-proBNP, pg/mL	777	1,259.5 (353.8–2,896.2)	922 (237–2,520)	2,045.5 (952.5–3,485.0)	874 (355.5–2,414.5)	0.056
Sodium, mEq/L	0	137 (135–139)	137 (135–139)	137 (135–139)	137.5 (135–139)	0.675
Potassium, mEq/L	0	4 (3.7–4.4)	3.9 (3.6–4.2)	4.1 (3.7–4.4)	4 (3.7–4.4)	0.003
Creatinine, mg/dL	0	1.2 (0.9–1.6)	1.1 (0.9–1.5)	1.2 (1.0–1.6)	1.1 (0.9–1.5)	0.101
eGFR, mL/min/1.73 m^2^	39	56 (45–60)	57 (48–67)	56 (43.5–60)	55 (44–60)	0.182
Echocardiogram						
LVEF, %	0	30 (25–35)	30 (20–35)	30 (25–40)	30 (25–35)	<0.001
IVSd, cm	1	1.2 (1.0–1.3)	1.1 (1.0–1.3)	1.2 (1.0–1.3)	1.2 (1.0–1.3)	0.140
LVIDd, cm	2	5.7 (5.1–6.3)	5.7 (5.1–6.3)	5.6 (5.1–6.3)	5.6 (5.1–6.2)	0.376
LVIDs, cm	6	4.8 (4.1–5.5)	4.9 (4.2–5.6)	4.7 (4.1–5.5)	4.7 (4.1–5.3)	0.203
PASP, mmhg	56	41.8 (31.7–50.2)	42.6 (31.8–50.1)	42.5 (33.0–51.2)	38.9 (29.7–49.4)	0.113
PW, cm	2	1.1 (1.0–1.3)	1.1 (1.0–1.3)	1.1 (1.0–1.3)	1.1 (1.0–1.3)	0.195
TAPSE, cm	19	1.7 (1.4–2.1)	1.7 (1.3–2.1)	1.7 (1.4–2.0)	1.8 (1.5–2.1)	0.046
Electrocardiogram						
QTc, ms	8	500 (474–531)	497 (474.8–530.2)	503 (476.0–534.8)	499 (471–528)	0.203
GDMT & target dose^Δ^						
RASi^ş^	147					0.022
none		139 (18.8)	47 (18.6)	57 (22.5)	35 (15.0)	
<50% target		309 (41.8)	97 (38.3)	116 (45.8)	96 (41.0)	
≥50% target		292 (39.5)	109 (43.1)	80 (31.6)	103 (44.0)	
*β*-Blocker	147					0.061
none		270 (36.5)	85 (33.6)	83 (32.8)	102 (43.6)	
<50% target		247 (33.4)	82 (32.4)	93 (36.8)	72 (30.8)	
≥50% target		223 (30.1)	86 (34.0)	77 (30.4)	60 (25.6)	
MRA	147					0.057
none		455 (61.5)	143 (56.5)	155 (61.3)	157 (67.1)	
<50% target		0	0	0		
≥50% target		285 (38.5)	110 (43.5)	98 (38.7)	77 (32.9)	
SGLT2i	147	62 (8.4)	26 (10.3)	21 (8.3)	15 (6.4)	0.306
GDMT Triple therapy	147	149 (20.1)	60 (23.7)	50 (19.8)	39 (16.7)	0.150
GDMT Triple therapy, ≥50% target	147	40 (5.4)	21 (8.3)	9 (3.6)	10 (4.3)	0.040
Loop diuretics	0	866 (97.6)	290 (98.3)	290 (98.0)	286 (96.6)	0.362

BMI, body mass index; BNP, B-type natriuretic peptide; BP, blood pressure; eGFR, estimated glomerular filtration rate; GDMT, guideline-directed medical therapy; IVSd, interventricular septum thickness at end-diastole; LVEDd, left ventricle end diastolic diameter; LVEF, left ventricular ejection fraction; LVIDd, left ventricular internal dimension at end -diastole; LVIDs, left internal dimension at end -systole; MAP, mean arterial pressure; MRA, mineralocorticoid receptor antagonist; NT-proBNP, N-terminal (NT)-pro hormone BNP; PASP, pulmonary artery systolic pressure; PW, left ventricular posterior wall; QTc, QT corrected for heart rate; RASi, renin-angiotensin-system inhibitor; SGLT2i, sodium glucose co-transporter 2 inhibitors; TAPSE, tricuspid annular plane systolic excursion.

* *p*-values comparing all BNP tertile groups using Chi-squared or Kruskal–Wallis.

Δ Target doses of guideline directed medical therapy (GDMT) as recommended in treatment guidelines.

ş RASi consisted of angiotensin-converting enzyme inhibitor, angiotensin receptor blocker, angiotensin receptor neprilysin inhibitor.

**Table 2:** GDMT use and tertile BNP tertile changes, multinomial logistic regression.

GDMT	Percent change tertiles vs. minimal BNP changes (reference)	OR	95% CI	*P*
RASi	Decreasing BNP tertile vs. reference	0.84	0.53–1.34	0.47
	Increasing BNP tertile vs. reference	0.63	0.39–1.02	0.06
β-Blocker	Decreasing BNP tertile vs. reference	1.16	0.78–1.73	0.47
	Increasing BNP tertile vs. reference	1.68	1.13–2.49	0.01
MRA	Decreasing BNP tertile vs. reference	0.83	0.57–1.22	0.35
	Increasing BNP tertile vs. reference	1.27	0.86–1.88	0.23

A multinomial logistic regression analysis was adjusted for potential factors influencing GTMT use included baseline age, sex, blood pressure, heart rate, atrial fibrillation, hypertension, creatinine and K+.

GDMT, guideline-directed medical therapy; RASi, renin-angiotensin-system inhibitor; MRA, mineralocorticoid receptor antagonist; OR, odds ratio; BNP, B-type natriuretic peptide.

The decreasing BNP group had elevated BNP levels, a median of 837.0 pg/mL (IQR 419.0–1,553.0) when compared to the other tertiles (*p* < 0.001). Potassium levels were elevated in the minimal changes and rising BNP tertiles (*p* = 0.003). Minimal changes and rising BNP tertiles had a higher LVEF. Baseline TAPSE (tricuspid annular plane systolic excursion) was also higher in the rising BNP tertile (*p* = 0.046).

The use of GDMT analysis showed that RASi were prescribed more frequently in the rising BNP group. However, the decreasing BNP group had a higher use of GDMT triple therapy at ≥50% target doses (8.3%, *p* = 0.040). The exploratory multivariate analysis resulted in Beta-blocker and increasing BNP levels [odds ratio [OR] 1.68; 95% confidence interval [CI] 1.13–2.49, *p* = 0.01]. Less RASi use and increasing BNP group (OR 0.63, 95% CI: 0.39–1.02, *p* = 0.06). The MRA therapy use did not differ (OR: 0.86–1.88, *p* = 0.23).

### Primary outcomes: changes in LV geometry and function

Linear mixed models for LVIDd different trajectories between tertiles (*p* = 0.006). The decreasing BNP group demonstrated a steady decrease in LVIDd, Figure 2B. Similarly, LVIDs steadily decreased in the decreasing BNP tertile (*p* < 0.001), Figure 2C. Finally, the decreasing BNP tertile exhibited an improvement in LVEF over time (*p* = 0.008), Figure 2D.

### Secondary outcome: all-cause mortality

**Figure 3:**
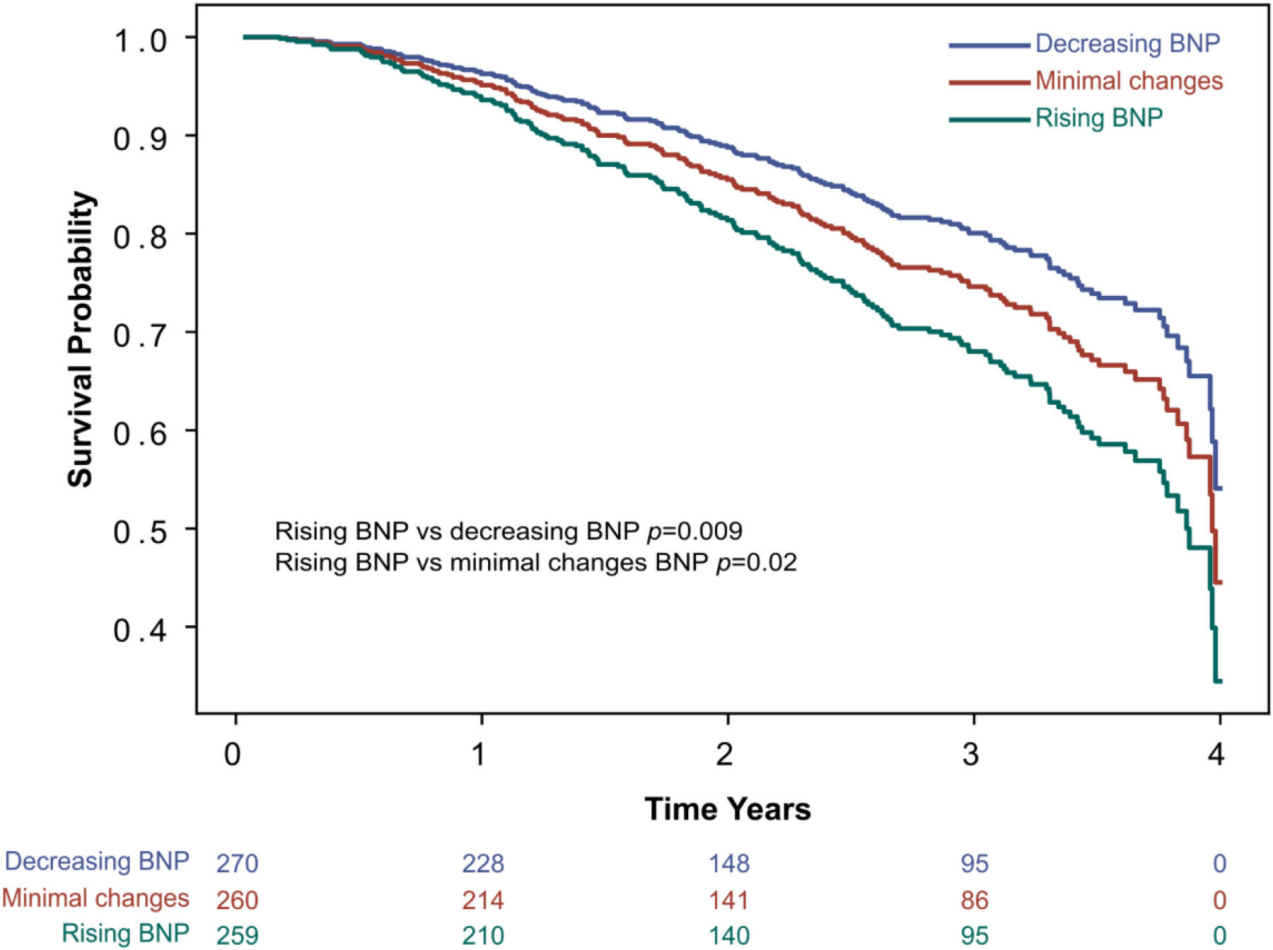
Secondary endpoint: all-cause mortality. Survival curve for all-cause mortality by BNP tertile change groups, adjusted for covariates in a Cox regression model. The Cox regression model was adjusted for potential clinically relevant covariates affecting BNP levels, including age in years, sex, BMI, atrial fibrillation, baseline BNP, creatinine, and LVEF. BMI, body mass index; BNP, brain natriuretic peptide; LVEF, left ventricular ejection fraction.

Cox regression analysis adjusted for clinically relevant covariates resulted in worse survival outcomes for the rising BNP tertile when compared to decreasing BNP (*p* = 0.009) and minimal BNP changes (*p* = 0.02), see Figure 3. Clinically relevant covariates included age, years, sex, BMI, heart rate, atrial fibrillation, baseline BNP, creatinine, and LVEF.

## Discussion

In this retrospective cohort, the results suggest an association between one-year changes in BNP levels and long-term LV remodeling in patients with HFrEF. Specifically, patients who experienced early reductions in BNP levels demonstrated favorable long-term LV remodeling and improved overall mortality outcomes. Findings underscore BNP changes as a dynamic marker for LV function.

### Cohort characteristics and BNP tertile interpretation

Baseline characteristics in Table 1, provide insights into the varying profiles across BNP tertiles. Compared to other groups, the decreasing BNP group comprised younger, higher proportion of females, and Hispanics, and higher blood pressure and heart rate values. In addition, it presented the lowest atrial fibrillation rates. In terms of HF severity, the decreasing BNP tertile had higher BNP levels at baseline, worse LVEF, and lower TAPSE compared to other groups. Finally, the use of RASi and GDMT Triple therapy at ≥50% was higher in this group. Taken together, the decreasing BNP group is indicative of younger patients with more severe HF but a better compensatory state and/or greater responsiveness to treatment (13).

The multivariate regression comparing BNP change tertiles and GDMT was adjusted for known factors that may affect GDMT use (Table 2). Beta-blocker use was significantly associated with the increasing BNP tertile, possibly reflecting more severe disease or symptomatic disease requiring therapy. Less RASi use presented borderline association with increasing BNP, suggesting possible underuse in more advanced heart failure. The MRA therapy use did not differ across groups.

### Interpretation of primary and secondary endpoints

The outcome analysis supports the hypothesis that BNP reductions at one-year is associated with favorable long-term LV remodeling and overall survival. The decreasing BNP tertile, characterized by substantial reductions in BNP from the indexation cohort date, showed significantly better LVIDd, LVIDs, and LVEF trajectories. This suggests that BNP changes may serve as an early predictive marker for long-term LV function. These findings are consistent with prior studies in myocardial infarction cohorts, where BNP has been shown to predict LV remodeling over time (6, 14).

BNP is a cardiac natriuretic peptide hormone primarily produced by ventricular myocytes. The primary stimulus for synthesis and secretion is myocyte stretch. The biological effects include diuresis, natriuresis, vasodilatation, inhibition of renin and aldosterone production, and regulation of cardiac and vascular myocyte growth (15). BNP acts as an endogenous brake on signaling pathways that drive the progression from LV hypertrophy through remodeling, heart failure, and death (16). Multiple studies have demonstrated BNP's anti-hypertrophic effects in both exogenous and endogenous experimental settings (17, 18).

Prior studies have shown that BNP levels can be valuable for prognosis. One study found that in a cohort of MI survivors over six months, patients with LV remodeling had higher levels of BNP on days 7, 90, and 180 compared to those without LV remodeling (19). Another study reported that in a cohort of MI survivors over one year, N-BNP levels were higher in patients with larger myocardial scars, which are identified as a major determinant of LV remodeling (20). While the relationship of LV remodeling and BNP is well established (6), long term longitudinal studies with serial imaging and biomarker data are limited due to the complexity and effort of longitudinal studies. Most relevant studies are short to mid term 6 to 12 months from secondary analyses (9, 21, 22). Our findings contribute to shading light on long term remodeling and BNP presenting 5 years of imaging and BNP relationships. HF is a dynamic disease, is important to characterize the dynamic changes and trajectories vs. as highlighted elsewhere (23–25).

Due to the mechanisms triggering BNP production and its biological effects, elevated BNP levels can reflect both LV systolic dysfunction and the body's compensatory response (26). These findings suggest that a BNP downtrend may be associated with improved systolic function, leading to a decrease in LV end-diastolic volume, reduced LV wall stress, and diminished BNP production. This can explain why patients with greater BNP reductions over the course of a year exhibited decreased LVIDs, lower LVIDd, and increased LVEF, which can be surrogate signs of LV remodeling improvement. Improved BNP and LV function directly translates into better survival outcomes.

### Clinical implications

In this study, we demonstrated that patients in the low BNP tertile had reduced LVIDs and LVIDd and increased LVEF compared to those in the high BNP tertile. These findings suggest that BNP dynamic reduction is indicative of LV remodeling improvement in a time-dependent fashion. Therefore, contributing to the literature of the relationship of BNP and LV remodeling dynamics.

### Study limitations

Limitations should be considered, the findings may not be generalizable to other populations due the single-center retrospective design. In addition, findings should be interpreted as associations rather than causal relationships. Cohort identification required follow-up BNP data, which may select more compliant or healthier patients, an inherent limitation of observational studies. While other factors during follow up can influence BNP or LV such as GDMT titration, adherence and device therapy, this was not explored. Limited NR-proBNP data available, due to system and provider causes most likely. Cause-specific mortality data was not collected in our institution, cause specific mortality analysis was not feasible.

## Conclusion

In a real-world routine HFrEF cohort, his study suggests that BNP changes within one year are associated with LV remodeling dynamics in the long term. Greater BNP reductions were associated with more favorable LV function, structural remodeling, and better all-cause mortality. Findings contribute to the growing literature of BNP as a dynamic marker for LV remodeling.

## Data Availability

The data analyzed in this study is subject to the following licenses/restrictions: IRB approval was made for data collection analysis and publication, however, sharing data outside of the UC Davis team was not granted. Requests to access these datasets should be directed to MC, mcadeiras@ucdavis.edu.
